# Step-by-step: the CD8 T cell journey in leishmaniasis

**DOI:** 10.1128/mbio.03537-23

**Published:** 2025-09-15

**Authors:** Erin A. Fowler, Fernanda O. Novais

**Affiliations:** 1 Department of Microbial Infection and Immunity, College of Medicine, The Ohio State University 683676 , Columbus, Ohio, USA; Instituto Carlos Chagas, Curitiba, Brazil

**Keywords:** CD8 T cells, parasites, *Leishmania*

## Abstract

Leishmaniasis is a group of vector-borne diseases caused by protozoan parasites of the genus *Leishmania* that affect millions of people across nearly 100 countries. Clinical presentations range from self-resolving cutaneous ulcers to life-threatening visceral disease, depending on the infecting species and host immune response. While CD4 T cells have long been recognized as central to parasite control, CD8 T cells also play an important role in disease. In this mini-review, we explore the many steps involved in CD8 T cell responses in leishmaniasis, with a focus on antigen recognition, recruitment, effector function, memory development, and dysfunction. Drawing on both murine models and human studies, we highlight the duality of CD8 T cells, which can contribute to protection but also drive immune-mediated tissue damage. Recent advances in transcriptomics, *in vivo* modeling, and immunophenotyping have begun to clarify the conditions under which CD8 T cells support vs hinder host defense. Despite this progress, critical questions remain, and continued investigation into the heterogeneity and regulation of CD8 T cells in leishmaniasis promises not only to deepen our understanding of host-pathogen interactions but also to guide the development of targeted immunotherapies and vaccines.

## INTRODUCTION

### Leishmaniasis

Leishmaniases are a spectrum of neglected tropical diseases caused by protozoan parasites of the genus *Leishmania*, transmitted through the bite of infected female sandflies (1). These parasites primarily reside and replicate within macrophages but can also infect a variety of innate immune cells (2). Leishmaniasis is endemic in 99 countries across Asia, Africa, the Americas, and parts of southern Europe, with more than 12 million people currently infected and approximately 1 million new cases reported each year (https://www.who.int/news-room/fact-sheets/detail/leishmaniasis). The disease burden is highest in low-resource settings, and control is complicated by rising drug resistance, treatment toxicity, and the absence of a licensed vaccine for use in humans.

The clinical manifestations of leishmaniasis are diverse and largely dictated by the infecting *Leishmania* species, host immune status, and environmental factors (2). The three major clinical forms are (i) cutaneous leishmaniasis, which causes localized skin ulcers, and a rare form, called diffuse cutaneous leishmaniasis, is associated with the spread of non-ulcerated skin lesions with uncontrolled parasite growth; (ii) mucosal leishmaniasis, in which parasites metastasize to and destroy mucosal tissues; and (iii) visceral leishmaniasis, the most severe form, which leads to systemic infection of the spleen, liver, and bone marrow and is uniformly fatal if untreated. Post-kala-azar dermal leishmaniasis is a complication that develops in a subset of patients who have recovered from visceral leishmaniasis, characterized by cutaneous macular or papulonodular lesions (3). Over 20 *Leishmania* species are known to infect humans (1). For instance, *L. major*, *L. tropica*, and *L. mexicana* typically cause cutaneous lesions, while *L. braziliensis* is associated with both cutaneous and mucosal disease. *L. donovani* and *L. infantum* are the primary agents of visceral leishmaniasis. Despite this diversity, all species share a common life cycle that includes extracellular promastigotes in the sandfly vector and intracellular amastigotes within mammalian hosts.

Control of *Leishmania* infection relies heavily on cellular immunity. CD4 T cells producing interferon-gamma (IFN-γ) are critical for parasite control, as IFN-γ activates infected macrophages to produce nitric oxide and reactive oxygen species that kill intracellular amastigotes (2). In contrast, the role of CD8 T cells is more complex and varies by disease form and tissue compartment. While CD8 T cells can contribute to parasite control in visceral leishmaniasis, in cutaneous disease, they are often implicated in tissue pathology. This review focuses on findings reported within the past 25 years using mouse models and human leishmaniasis to provide a broader understanding of how CD8 T cells respond in different clinical presentations.

### CD8 T cells

CD8 T cells are a critical arm of the adaptive immune system, specialized in identifying and eliminating infected or abnormal cells. These cells recognize antigenic peptides presented by major histocompatibility complex (MHC) class I molecules via their T cell receptor (TCR), and upon activation, they differentiate into effector T cells capable of producing inflammatory cytokines and cytotoxic molecules. Their activation requires not only TCR engagement but also co-stimulatory signals and cytokines provided by antigen-presenting cells. Once activated, CD8 T cells expand clonally, migrate to infected tissues, and exert a range of functions depending on the nature of the infection and the local microenvironment.

Chemokine gradients and receptor expression guide their recruitment to sites of infection, while the parasite antigens shape their activation and differentiation. In the context of *Leishmania* infection, CD8 T cells exhibit functional diversity, including the production of cytokines such as IFN-γ, TNF-α, IL-17, and IL-10, and the release of cytolytic granules containing perforin and granzymes. These effector functions can contribute to parasite control and protection from disease or promote tissue damage, and the context promoting one or the other will be discussed in detail. Following pathogen clearance or immunization, some CD8 T cells differentiate into memory subsets, which may circulate through lymphoid tissues or reside in peripheral tissues such as the skin. Tissue-resident memory T cells, in particular, are well positioned to respond rapidly to reinfection and may play a key role in long-term disease protection in barrier sites (4).

This mini-review explores the roles of CD8 T cells in leishmaniasis across key stages of their response, including antigen recognition, recruitment, effector activity, memory formation, and dysfunction, highlighting how parasite species, tissue environment, and disease form shape their contribution to immunity or pathology.

## STEP 1: ANTIGEN PRESENTATION TO CD8 T CELLS

Despite substantial evidence supporting a role for CD8 T cells in the immune response to *Leishmania*, the specific antigens they recognize remain unidentified. Multiple attempts to define immunodominant epitopes have been unsuccessful, and no dominant TCR clonotypes have emerged. This fundamental gap limits our ability to assess the antigen-specific CD8 T cell response during infection and complicates efforts to distinguish protective vs pathogenic subsets. The possibility that bystander CD8 T cells may be recruited to inflamed tissue and contribute to immunopathology further underscores the need for precise tools to study antigen recognition (see “Bystander activation” below).

**Fig 1:**
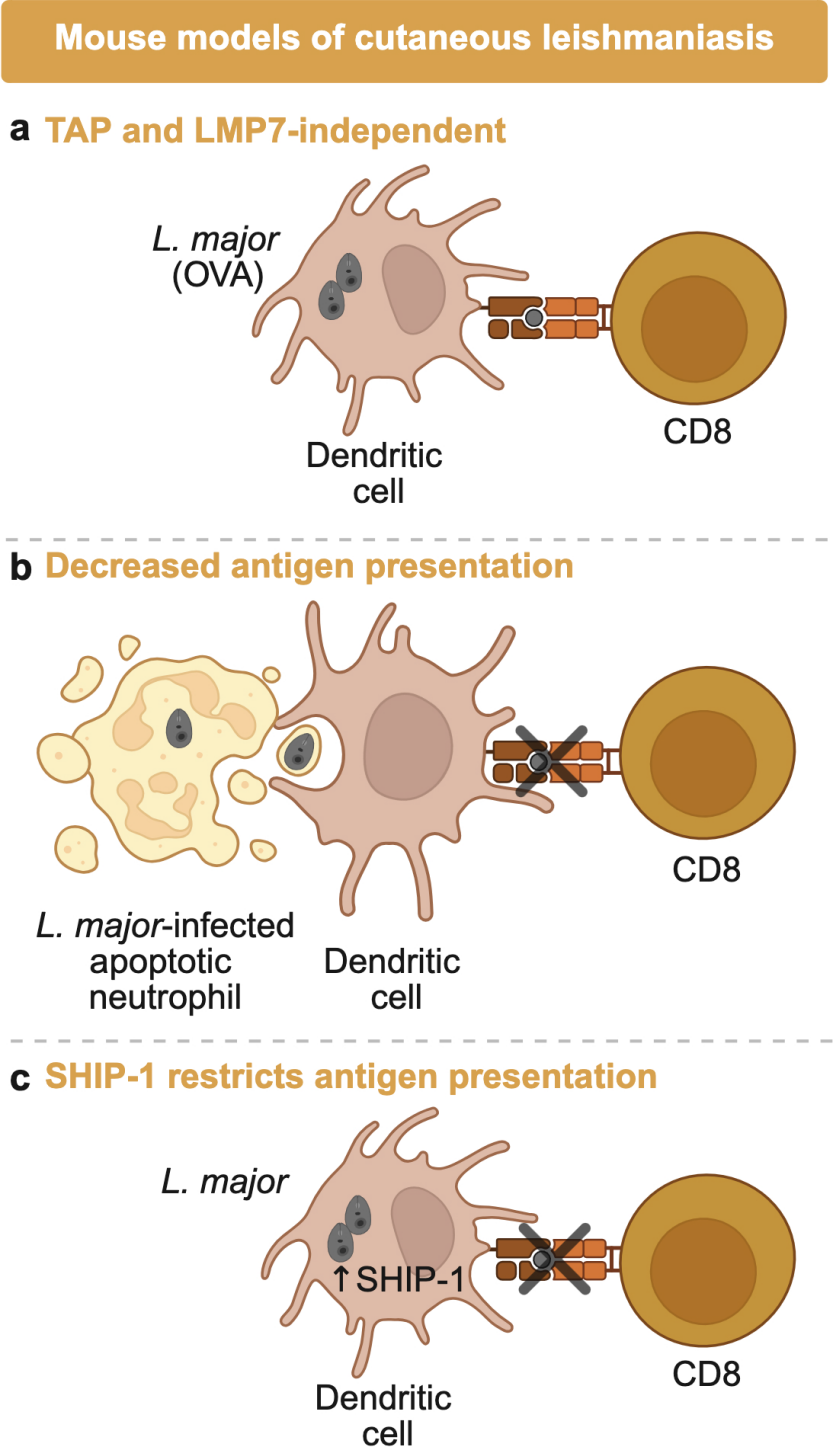
Antigen presentation to CD8 T cells in murine cutaneous leishmaniasis. Studies using ovalbumin-expressing *L. major* as a model for antigen presentation showed that the antigen processing of *Leishmania* is non-canonical compared to other intracellular pathogens, and these pathways remain unclear. (**a**) Typical antigen processing machinery, transporter associated with antigen processing pathway, and the immunoproteasome LMP7 are not required for dendritic cells to present antigen to OVA-specific CD8 T cells. Furthermore, *Leishmania* possesses various mechanisms to antagonize presentation by dendritic cells. (**b**) Apoptotic *L. major*-infected neutrophils are engulfed by dendritic cells, reducing antigen presentation by dendritic cells. (**c**) *L. major* has also been shown to inhibit SH2-containing inositol 5-phosphatase 1 (SHIP-1) in dendritic cells, which leads to reduced acidification of the *Leishmania*-containing endosome and subsequently less efficient antigen processing.

To overcome the challenge posed by unknown parasite epitopes, researchers have used *L. major* parasites engineered to express model antigens such as ovalbumin (OVA). These models have been instrumental in dissecting the rules governing antigen presentation to CD8 T cells. Studies using OVA-expressing parasites demonstrated that subcellular localization of antigen strongly influences the CD8 T cell response: secreted forms of OVA elicit more robust OT-I CD8 T cell proliferation compared to non-secreted versions (5, 6). These findings suggest that secreted parasite antigens are more efficiently processed and presented by antigen-presenting cells, likely because they are more accessible for cross-presentation. However, even when *Leishmania* antigens are secreted, the route of cross-presentation appears to differ from that used by other intracellular pathogens. Unlike *Mycobacterium tuberculosis* or *Listeria monocytogenes*, where cytosolic translocation and transporter associated with antigen processing (TAP)-dependent processing are typically required (7, 8), presentation of *Leishmania*-derived OVA antigens occurs independently of TAP. Bertholet and collaborators (9) demonstrated that CD8 T cell activation in response to OVA-secreting *L. major* was TAP independent both *in vitro* and *in vivo* (Fig. 1a). This suggests that *Leishmania* antigens may be processed and loaded onto MHC class I molecules directly in the phagosome or via peptide regurgitation onto the cell surface. Further supporting a non-canonical presentation pathway, priming of CD8 T cells *in vivo* also appears to be independent of the immunoproteasome (Fig. 1a), as mice lacking LMP7 (a subunit of the immunoproteasome) infected with *L. major* develop lesions and CD8 T cell responses comparable to wild-type controls (10).

Several host- and pathogen-driven mechanisms further complicate antigen presentation in leishmaniasis. For example, dendritic cells that engulf apoptotic neutrophils from *L. major*-infected lesions exhibit impaired cross-presentation of antigens to CD8 T cells (Fig. 1b). Notably, this inhibition is observed only when neutrophils are infected, potentially due to accelerated apoptosis and altered apoptotic signaling (11). Additionally, *L. major* actively impairs antigen processing by modulating host signaling pathways. The parasite induces activation of SH2-containing inositol 5-phosphatase 1 (SHIP-1) in dendritic cells, a phosphatase that restricts antigen presentation (Fig. 1c). Inhibition of SHIP-1 enhances endosomal acidification, improves antigen processing, and boosts CD8 T cell responses in vaccination models (12). These data suggest that *Leishmania* employs multiple strategies to interfere with antigen processing and presentation, likely as a means to evade CD8 T cell recognition.

Together, these findings underscore how little is known about the identity of antigens recognized by CD8 T cells during *Leishmania* infection and the pathways by which they are processed and presented. Although model systems have illuminated general principles of cross-presentation in leishmaniasis, the absence of defined parasite-derived epitopes continues to impede mechanistic understanding of antigen-specific CD8 T cell responses in both primary infection and vaccine settings.

## STEP 2: CD8 T CELL RECRUITMENT

To influence disease, CD8 T cells must first be recruited to the site of infection, which is primarily dictated by chemokine and chemokine receptor interactions. Cutaneous lesions from *L. braziliensis*-infected patients exhibit high expression of the CXCR3 ligands CXCL9 and CXCL10 (13). which are likely critical for recruitment, as CXCR3-deficient mice show impaired recruitment of both CD4 and CD8 T cells to *L. major*-infected lesions (14). These mice also exhibit poor parasite control and increased lesion pathology, underscoring the importance of this chemokine axis in protective immunity (14). However, because both T cell subsets are affected, the specific contribution of CD8 T cells to these outcomes remains unclear. Given the well-established requirement for CD4 T cells in parasite control, it is likely that the defective CD4 T cell recruitment plays a dominant role in the increased susceptibility observed in CXCR3-deficient animals. In mouse models of *L. donovani* infection, CXCL10 is critical for attracting CD8 T cells following vaccination (15), supporting a role for CXCR3 in the recruitment of CD8 T cells in leishmaniasis.

Other chemokine pathways have also been implicated in CD8 T cell recruitment. In *L. braziliensis*-infected human skin, CCR5 is the most highly expressed chemokine receptor transcript relative to healthy skin (16). Its ligands, CCL3 and CCL4, are associated with prolonged healing times in patients, suggesting a potential link between CCR5-driven recruitment and disease persistence (16). Supporting this, CCR5 deficiency in CD8 T cells reduces their accumulation in lesions caused by *L. braziliensis* and attenuates pathology in mouse models (16). In post-kala-azar dermal leishmaniasis, caused by *L. donovani*, CD8 T cells are the dominant T cell subset in the dermis and exhibit increased CCR4 expression (17). CCL17 and CCL22, ligands for CCR4, are highly expressed and decline after treatment, suggesting their important role (17). Furthermore, CD8 T cells from *L. braziliensis* patients reduce CCR7 expression and increase cutaneous lymphocyte-associated antigen expression following antigen restimulation (18), which supports their recruitment to the inflamed skin.

In cutaneous leishmaniasis caused by *L. braziliensis*, the composition of T cells within lesions shifts as disease progresses. Notably, CD8 T cells become more abundant in advanced lesions, while the frequency of CD4 T cells remains relatively constant (19). This shift in the CD4:CD8 ratio is accompanied by increased granzyme A expression among CD8 T cells (19), suggesting that these cells may play a pathogenic role in disease exacerbation rather than protection from disease. In support, a recent study using a controlled human infection model of sandfly-transmitted *L. major* parasites, which is both elegant and challenging to accomplish, showed a high frequency of CD8 T cells in lesions, particularly in recurrent or late-stage lesions (20). While these observations indicate that CD8 T cells accumulate as cutaneous lesions worsen, they do not establish whether their presence and/or increased cytotoxicity is a cause or consequence of disease severity—a distinction that has been established by other studies (see “Granule-mediated cytotoxicity” below).

**Fig 2:**
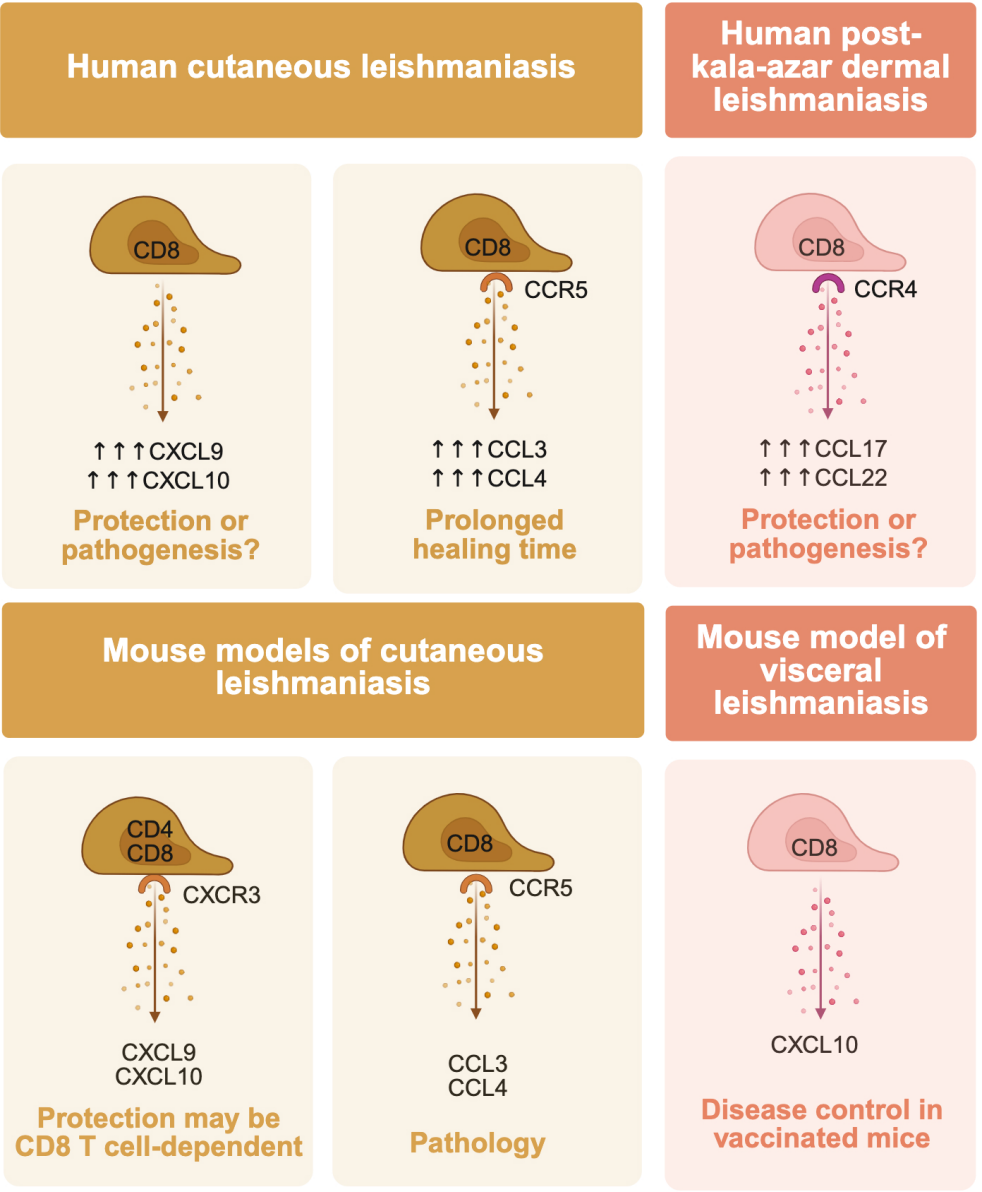
CD8 T cell recruitment and its role in protection or pathogenesis in leishmaniasis. In all forms of leishmaniasis, CD8 T cells are recruited to the site of infection by various T cell-attracting chemokine gradients. Cutaneous leishmaniasis: in the lesions of patients, there is high expression of the CXCR3 ligands CXCL9 and CXCL10, as well as the chemokine receptor CCR5 and its ligands CCL3 and CCL4. CCR5 expression in the lesions of patients corresponds to longer lesion healing time, and, in agreement, murine models of the disease illustrated that CCR5 signaling recruits pathogenic CD8 T cells to skin lesions. In mice, CXCL9/10 signaling through CXCR3 corresponds to increased parasite numbers and pathology, but the recruitment of both CD4 T cells and CD8 T cells was impaired. Due to this, the contributions of CXCR3 signaling on CD8 T cell-mediated pathology vs the recruitment of protective CD4 T cells are uncertain. Visceral leishmaniasis: unlike cutaneous disease, CD8 T cells are generally considered protective in this clinical manifestation. In mouse models, CXCL10 expression after vaccination confirmed the chemokine’s role in recruiting CD8 T cells to sites of *Leishmania* infection, and the presence of CD8 T cells corresponded to protection. In human post-kala-azar dermal leishmaniasis, the role of CD8 T cells in protection and pathology is unclear. However, they are the major T cell subset within the lesions, and their presence corresponds to an increase in the expression of CCR4 and its ligands CCL17 and CCL22.

In summary (Fig. 2), multiple chemokines and chemokine receptors can be involved in the recruitment of CD8 T cells to the skin, and their abundance in the tissue, and particularly the ratio between CD4 and CD8 T cells in the tissue, can inform disease outcomes.

## STEP 3: EFFECTOR FUNCTIONS—CD8 T CELLS IN PRIMARY INFECTION

### Cytokine production

CD8 T cells are capable of producing a range of cytokines during *Leishmania* infection, although the context and anatomical site critically shape their contributions. In early studies using low-dose *L. major* infection models, CD8 T cell-derived IFN-γ was shown to be necessary for optimal induction of protective Th1 responses, particularly through support of IL-12-driven CD4 T cell polarization (21–23). However, in high-dose infection models, early IFN-γ production by CD8 T cells was not required to generate sufficient Th1 responses, and their contribution became dispensable (22). Consistent with these findings, CD8 T cells produce IFN-γ in draining lymph nodes but very little within lesional skin (21, 24), where they appear to adopt a different phenotype (25). In human *L. braziliensis* infection, IFN-γ production is primarily attributed to CD4 T cells rather than CD8 T cells (18, 26). Similarly, in *L. major*-infected patients, peripheral CD8 T cells produce IFN-γ *ex vivo*, although at significantly lower levels than CD4 T cells (27). These observations suggest that while CD8 T cells may transiently contribute to IFN-γ production early during infection, especially in lymphoid tissues, they are not the primary source of this cytokine at the effector site.

Beyond IFN-γ, CD8 T cells have also been implicated in the production of IL-17 in mucosal leishmaniasis. IL-17-producing CD8 T cells are found in lesions from mucosal patients (28), and in murine models of cutaneous leishmaniasis, IL-17-deficient CD8 T cells show delayed kinetics in lesion development, though they ultimately develop severe lesions upon *L. braziliensis* infection (29). These data suggest that IL-17 from CD8 T cells may contribute to early inflammatory responses but is insufficient to drive protective immunity. Taken together, cytokine production by CD8 T cells is spatially regulated, but our understanding remains very limited and insufficient to explain either disease control or pathology in most models of *Leishmania* infection.

### Granule-mediated cytotoxicity

The primary mechanism used by CD8 T cells to eliminate intracellular pathogens and tumor cells is granule-mediated cytotoxicity (30). This pathway is initiated when CD8 T cells recognize antigen-presenting target cells and form an immunological synapse, through which they release cytotoxic granules containing perforin and granzymes. Perforin is a pore-forming protein that inserts into the target cell membrane, facilitating the delivery of granzymes, a family of serine proteases that enter the cytosol through these pores. Once inside, granzymes trigger programmed cell death by cleaving intracellular substrates, activating caspases, and inducing mitochondrial damage, leading to apoptosis. While granule-mediated cytotoxicity is essential for immune defense against infections and malignancies, dysregulated or excessive cytotoxic responses can result in tissue damage and pathological inflammation. Indeed, the cytotoxic activity of CD8 T cells has been implicated in the pathology of various inflammatory skin diseases, including autoimmune alopecia, vitiligo, bullous pemphigoid, Stevens-Johnson syndrome, and toxic epidermal necrolysis (31–36).

The cytotoxic function of CD8 T cells has been studied extensively, especially in the context of cutaneous leishmaniasis. While cytotoxic CD8 T cells can be detected in the blood and lesions of patients across several forms of the disease, their functional role varies considerably. In *L. panamensis* infection, patients with active disease exhibit significantly more CD8 T cells expressing granzyme B and perforin compared to those who are cured (37). In contrast, CD8 T cells in post-kala-azar dermal leishmaniasis lesions from *L. donovani*-infected individuals express granzyme B and perforin at levels similar to those in healthy skin (17), and in diffuse cutaneous leishmaniasis caused by *L. mexicana*, CD8 T cells fail to kill infected autologous macrophages (38). Granzyme A, another cytotoxic effector molecule, is elevated in mucosal leishmaniasis caused by *L. braziliensis* compared to cutaneous lesions (39), supporting a potential association between cytotoxicity and disease severity. In visceral leishmaniasis, CD8 T cells from the blood of patients express perforin and granzyme A, but treatment appears to decrease their expression only in peripheral compartments, not in splenic tissue (40). Overall, the role of cytotoxicity in CD8 T cells does not support parasite control and is associated with more severe disease, particularly in the cutaneous forms of leishmaniasis.

The most mechanistically detailed body of work comes from studies of *L. braziliensis* infection, where cytotoxic CD8 T cells contribute to pathology rather than parasite control and disease protection. In both patients and mouse models, lesional CD8 T cells express high levels of perforin and granzyme B (13, 18, 25, 29, 41–44), yet their cytotoxic activity does not correlate with parasite control *in vitro* or *in vivo*. In fact, adoptive transfer of CD8 T cells into T cell-deficient mice results in severe skin damage, an effect abrogated when the transferred CD8 T cells lack perforin (29). Notably, while cytotoxic killing did not aid in parasite clearance, it promoted parasite dissemination to distant tissues. These findings suggest that granule-mediated cytotoxicity facilitates tissue damage and metastasis rather than resolving infection. Mechanistic studies further revealed that CD8 T cell-induced cell death activates the NLRP3 inflammasome, leading to IL-1β release and sustained neutrophil recruitment (43). mRNA sequencing of patient lesions confirmed that higher expression of cytotoxic and inflammasome-related genes correlates with delayed healing despite anti-parasitic treatment, reinforcing the idea that cytotoxic CD8 T cells drive immunopathology in cutaneous disease (41). Based on these findings, targeted immunotherapies are being explored. For example, a topical perforin inhibitor recently showed efficacy in reducing skin damage in mouse models (44), raising the possibility of combinatorial therapies that modulate immune-mediated pathology in the presence of anti-parasitic drugs.

**Fig 3:**
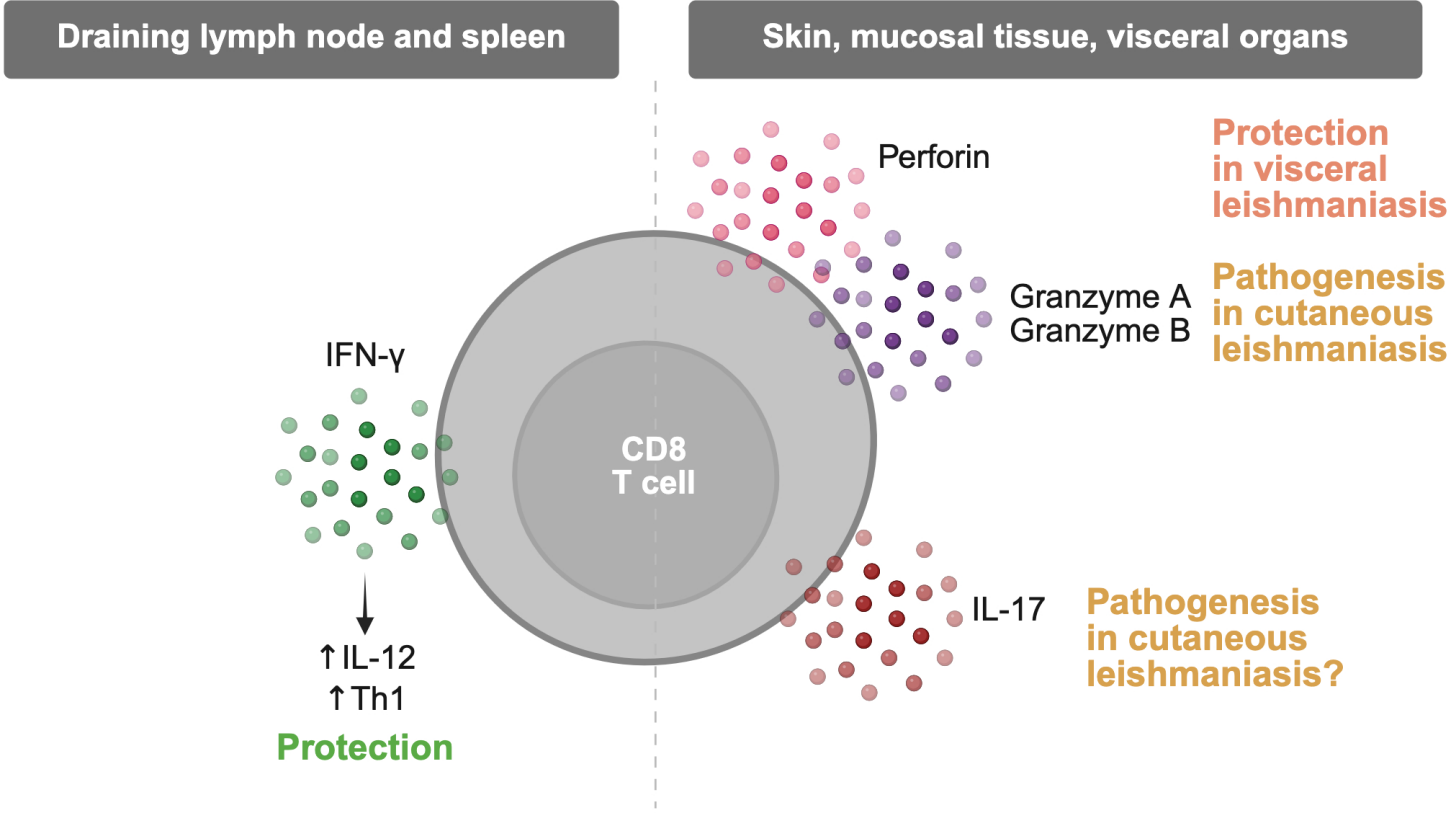
The effector functions of CD8 T cells and their role in protection and tissue damage in leishmaniasis. CD8 T cells possess numerous effector functions, but three main functions have been the most extensively studied in the context of leishmaniasis. The production of the cytokine IFN-γ is associated with protection in all forms of the disease. When IFN-γ is expressed in the draining lymph nodes, CD8 T cells promote the production of the Th1 polarizing cytokine IL-12, enhancing the formation of protective Th1 CD4 T cells. The production of IL-17 has been described within the lesions of mucosal leishmaniasis, a severe form of cutaneous leishmaniasis, characterized by few parasites and pathology associated with an overactive T cell response. In a murine model of cutaneous leishmaniasis, IL-17 deficiency in CD8 T cells slowed lesion progression, indicating a potential role in the pathology of these forms of the disease. Lastly, cytotoxic activity appears to have paradoxical roles depending on the clinical presentation. Production of the cytotoxic molecules in the spleen during visceral disease is associated with protection. However, perforin and the granzymes A and B are elevated in the lesions of cutaneous and mucosal leishmaniasis patients and correspond to disease severity. Mouse models of cutaneous disease have further illustrated the strong connection between the presence of cytotoxic CD8 T cells and pathology.

In summary (Fig. 3), CD8 T cells display diverse effector functions during *Leishmania* infection, shaped by tissue localization, parasite species, and disease form. In cutaneous leishmaniasis, cytokine production by CD8 T cells, particularly IFN-γ, is largely limited to early responses in lymphoid tissues, with little evidence of functional cytokine production within lesions. In contrast, cytotoxic activity is a prominent feature in the skin, where CD8 T cells expressing perforin and granzymes contribute to immunopathology rather than parasite control. It is possible that in patients infected with *L. amazonensis*, for example, in which lesions have an abundance of infected cells and many fewer T cells, this dichotomy may be less relevant. In support, NLRP3 inflammasome and IL-1β play a protective role in *L. amazonensis* infection (45), although inflammasome activation here may be unrelated to CD8 T cell cytotoxicity. Together, these findings underscore the context-dependent nature of CD8 T cell responses in leishmaniasis and support the development of targeted immunotherapies aimed at mitigating immunopathology in certain cutaneous forms.

## STEP 4: CD8 T CELLS IN SECONDARY INFECTION, VACCINATION, AND LONG-TERM IMMUNITY

**Fig 4:**
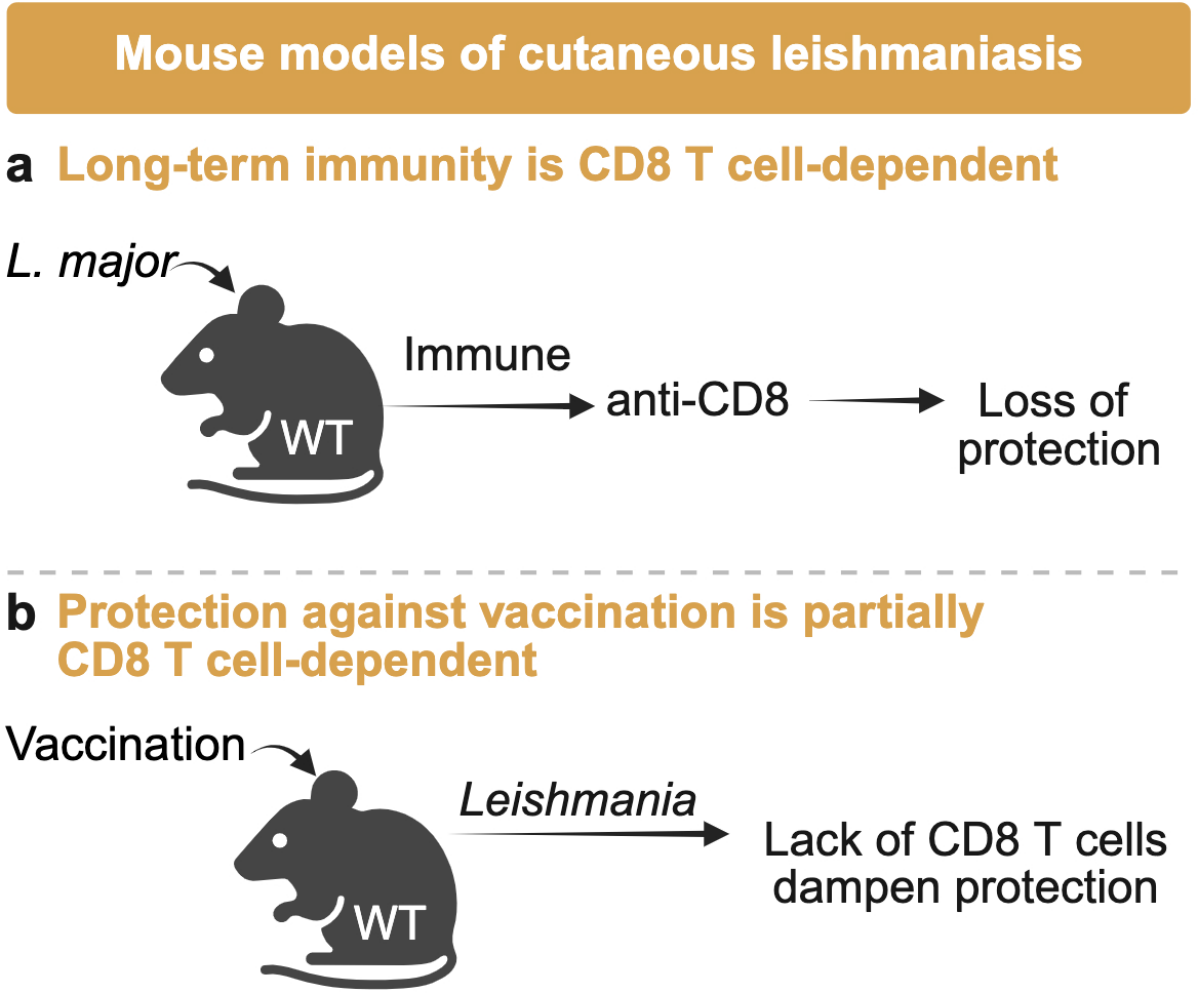
A role for CD8 T cells in long-term anti-*Leishmania* immunity. (**a**) In some mouse models of infection, treatment with an anti-CD8 T cell depleting antibody after lesion resolution led to a break in parasite control and the reoccurrence of skin pathology in wild-type (WT) mice. (**b**) Efficient immunity in various models of vaccination was dependent on the ability to present antigen to and the presence of CD8 T cells.

Although there are currently no licensed vaccines for human leishmaniasis, individuals and mice that resolve primary infection typically develop protection against secondary challenge, but the role of CD8 T cells is unclear. For example, Belkaid and colleagues (46) demonstrated that depletion of CD8 T cells 3 months after resolution of cutaneous lesions caused by *L. major* led to parasite reactivation and renewed skin pathology (Fig. 4a), while in another study, CD8 T cells were found to be dispensable for immunity to secondary challenge (47). The discrepancy between these findings is unknown and should be further investigated.

In contrast to infection-induced immunity, several studies show the importance of CD8 T cells in vaccine-induced immunity (Fig. 4b). For example, CD8 T cells from vaccinated animals produced IFN-γ in response to stimulation with *L. major*-infected dendritic cells, and *in vivo* depletion of CD8 T cells abrogated parasite control, demonstrating their necessity for effective vaccine-induced immunity (48). Similar results were observed in mice vaccinated against *L. amazonensis*, where β2-microglobulin-deficient mice, lacking MHC class I expression, showed diminished protection measured by more parasites and larger lesions in deficient mice compared to wild type (49). However, it remains unclear whether this phenotype reflects a defect in memory formation during vaccination or an essential role for CD8 T cells specifically during challenge. In visceral leishmaniasis models, CD8 T cells have consistently emerged as critical contributors to vaccine-induced protection. Vaccination with hydrophilic acylated surface protein B1 from *L. donovani* resulted in fewer parasites that depended on IL-4 produced by phagocytes to prime CD8 T cell responses (50). Similarly, CD8 T cell depletion increased parasite numbers in *L. major*-infected mice immunized with kinetoplastid membrane protein-11 (51) and the *Leishmania* homolog of the receptor for activated C kinase upon *L. donovani* infection (52). Control of *L. panamensis* challenge was also CD8 T cell dependent (53). Notably, however, not all vaccine-induced immunity requires CD8 T cells. In one study, immunization with nucleoside hydrolases from *L. donovani* provided protection against *L. amazonensis* infection independently of CD8 T cells, instead relying on CD4 T cells (54). Together, these findings highlight the diversity of immune mechanisms that can be leveraged by different vaccine platforms and suggest that CD8 T cells may be particularly important when antigens are presented in ways that favor cross-presentation.

While protection against secondary infection may not always require CD8 T cells, a growing body of evidence indicates that their role becomes more prominent in the context of experimental vaccination. As vaccine development for human leishmaniasis advances, understanding when and how CD8 T cells contribute to protective immunity will be critical for optimizing vaccine design and efficacy.

## THERE ARE MANY MORE STEPS TO CONSIDER

### Microenvironmental signals shaping CD8 T cell responses

Microenvironmental signals play a critical role in shaping immune cell function at sites of infection and inflammation (55). These signals include hypoxia, inflammatory cytokines, extracellular ATP, and other metabolic cues and can modulate cytotoxic potential, survival, and exhaustion, ultimately influencing disease outcome. Understanding how these local factors regulate immune responses is essential for identifying new strategies to enhance protective immunity while minimizing immunopathology.

Hypoxia, or low oxygen, occurs when the supply of oxygen fails to meet the tissue’s demand and is a defining feature of the cutaneous leishmaniasis lesion microenvironment (25, 56). For example, in cutaneous leishmaniasis, CD8 T cells upon recruitment from oxygen-rich draining lymph nodes encounter a hypoxic environment in the infected skin that triggers transcriptional and metabolic reprogramming (25). One key outcome is the upregulation of the transcriptional repressor Blimp-1, which drives terminal differentiation and enhances granzyme B expression (25). Interestingly, hypoxia also promotes perforin expression through a Blimp-1-independent mechanism, indicating that distinct regulatory pathways control different cytotoxic molecules (44). Beyond shaping CD8 T cell effector function directly, hypoxia also alters the broader cytokine landscape. For example, it has been shown to suppress IL-12 production (57), a cytokine crucial for IFN-γ production. This may help explain the observed dichotomy between high IL-12 levels in draining lymph nodes and low IL-12 levels in lesional skin (24), contributing to the emergence of functionally distinct CD8 T cell populations in these tissues.

Among the cytokines enriched in lesional skin, IL-15 plays a particularly important role in driving cytotoxicity (42). While IL-15 is classically associated with CD8 T cell homeostasis, it can also induce CD8 T cell effector functions. IL-15 alone is sufficient to trigger granzyme B and perforin expression in CD8 T cells and cooperates with hypoxia to amplify their cytotoxic potential (42, 44). In both mouse and human systems of cutaneous disease caused by *L. braziliensis* and *L. major*, IL-15 also upregulates the activating receptor NKG2D (58), a feature that promotes bystander CD8 T cell activation (see “Bystander activation” below). Together, the combined effects of hypoxia and IL-15 in the skin create a unique microenvironment that drives CD8 T cells toward terminal differentiation and cytotoxicity, traits often associated with immunopathology in cutaneous diseases caused by *L. braziliensis*.

The local cytokine milieu is further influenced by anatomical location and parasite species. In the draining lymph nodes, where IL-12 is more abundant and oxygen tension is higher, CD8 T cells adopt a more protective phenotype (24, 25, 44). In contrast, in the hypoxic, IL-15-rich skin, CD8 T cells often exhibit a pathogenic profile. These site-specific environmental cues may also vary between infections caused by different *Leishmania* species. For instance, *L. braziliensis* and *L. amazonensis* elicit distinct inflammatory responses (2), which likely shape the cytokine composition and modulate the requirements for CD8 T cell-mediated immunity.

**Fig 5:**
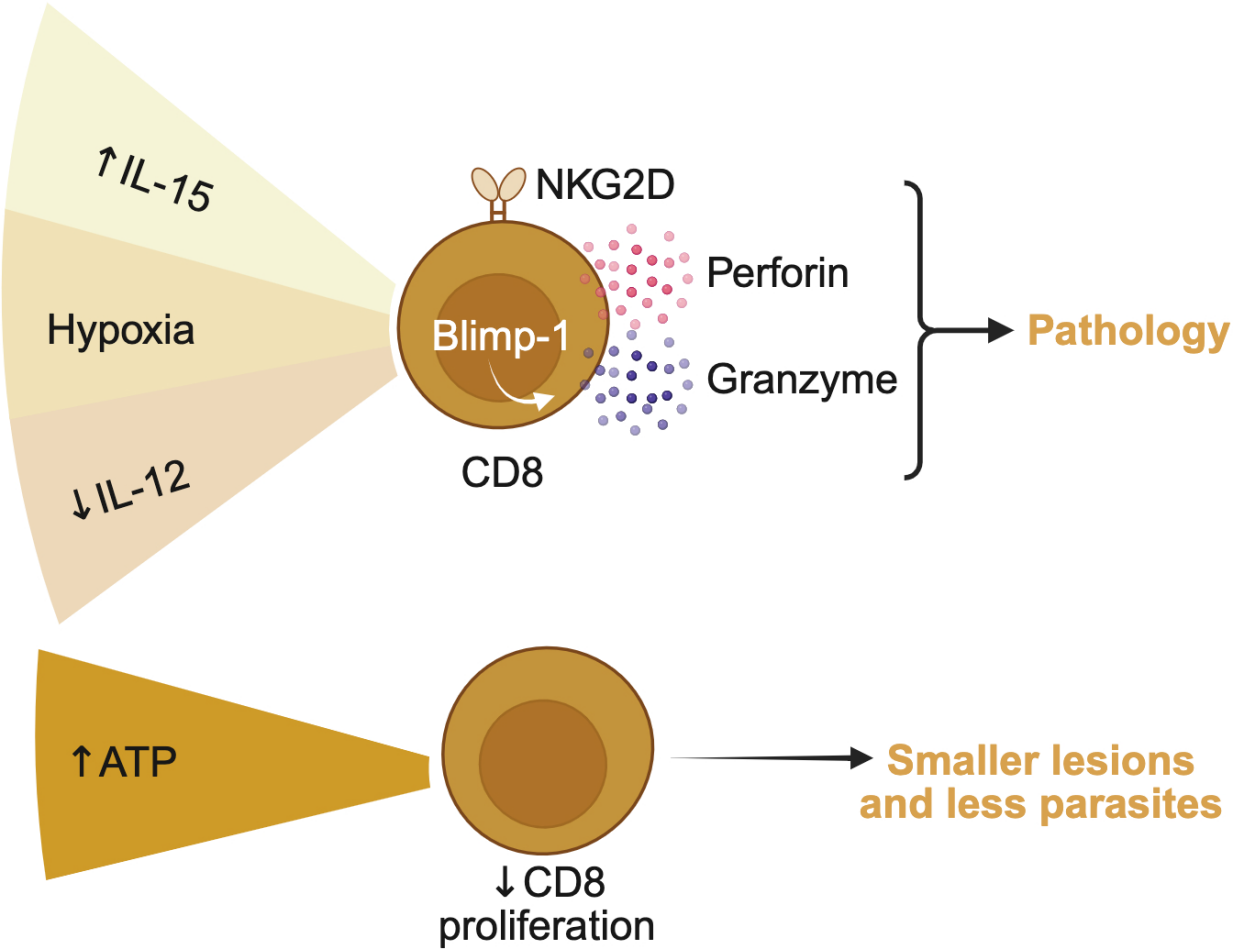
Microenvironmental signals impacting the effector function of CD8 T cells in cutaneous leishmaniasis. Cytotoxic CD8 T cells expressing granzymes and perforin are associated with pathology in cutaneous leishmaniasis, and many microenvironmental signals in the lesion have been shown to induce cytotoxic CD8 T cells. Skin lesions of patients and mice are hypoxic, which induces the expression of perforin and, via upregulation of the transcriptional repressor Blimp-1, granzyme B. IL-15 mRNA is significantly increased in the skin lesion of mice and humans, and IL-15 induces the expression of cytotoxic molecules by CD8 T cells and is further increased in cooperation with hypoxia. Furthermore, IL-15 also induces the expression of the activating receptor NKG2D, which is associated with the activation of pathogenic, bystander CD8 T cells. The cytokine IL-12 is associated with inducing the protective CD8 T cell response in the draining lymph nodes and, correspondingly, is found in low levels in the skin lesion. Hypoxia has been shown to reduce the expression of IL-12 by myeloid cells, likely contributing to the low levels of IL-12 in the lesions and preventing the development of IFN-γ-producing CD8 T cells. While hypoxia and cytokine milieu of the lesion promote the pathogenic phenotype, other factors like the signaling of extracellular ATP by CD8 T cells reduce their proliferation, leading to smaller lesions and lower parasite burden.

In addition to cytokines and oxygen tension, other environmental signals can regulate CD8 T cell responses. Extracellular ATP, released during inflammation, engages purinergic P2 receptors on immune cells and shapes cellular activation. Deficiency in the ATP receptor P2X7 leads to increased lesion size and parasite burden in *L. amazonensis*-infected mice, accompanied by enhanced CD8 T cell proliferation (59). While the direct impact of P2X7 on CD8 T cells is not fully defined, these findings suggest that ATP signaling may help restrain CD8 T cell expansion. Moreover, vector-derived factors also modulate lesion immunology. In experimental infections with *L. amazonensis*, sandfly salivary gland components increase IL-10 production by CD8 T cells (60), potentially dampening their effector functions and favoring parasite persistence. Overall (Fig. 5), these diverse signals influence the behavior of CD8 T cells in leishmaniasis, and considering the influence of the microenvironment is critical to understanding cell-fate decisions. Furthermore, other factors such as tissue pH and temperature (which is, for example, higher in visceral organs and lower in the skin) may further contribute to this variation. Their effects on CD8 T cell function in leishmaniasis remain largely unexplored.

### Bystander activation

Bystander activation—defined as the recruitment and activation of T cells that are not specific for the pathogen in question—has emerged as an important feature of CD8 T cell responses during *Leishmania* infection. In visceral leishmaniasis caused by *L. donovani*, parasite control is strictly dependent on CD8 T cells recognizing cognate antigen, and bystander CD8 T cells play no discernible protective role (61). In this context, antigen specificity appears to be a critical determinant of CD8 T cell efficacy, with little room for collateral or non-specific contributions. In contrast, in cutaneous leishmaniasis, bystander CD8 T cells appear to be not only present but also pathogenic (58, 62, 63). Studies have shown that CD8 T cells specific for unrelated pathogens, such as viruses, are present in lesions from *Leishmania*-infected patients (64). In mouse models, prior viral infection exacerbates disease severity upon subsequent *L. major* challenge, and this is associated with increased recruitment of virus-specific CD8 T cells to the infected skin (62). Co-infection with *L. major* and a virus leads to significantly larger lesions, a phenotype that can be reversed by CD8 T cell depletion (63). Mechanistically, these bystander CD8 T cells are activated by the inflammatory microenvironment and upregulate granzyme B and the activating receptor NKG2D (62, 63). NKG2D binds to stress ligands, such as Rae1 in mice and MICA/B in humans, that are upregulated in *Leishmania*-infected tissue. Engagement of NKG2D drives cytotoxic killing via granzyme B, contributing directly to tissue destruction. Notably, IL-15, which is enriched in cutaneous lesions from *L. braziliensis*-infected patients, promotes NKG2D expression on CD8 T cells, and blocking NKG2D signaling reduces CD8 T cell degranulation and cytotoxicity (58). These findings position IL-15 as a key amplifier of bystander-mediated tissue damage in the skin (Fig. 5).

While most evidence points to a detrimental role for bystander CD8 T cells in cutaneous disease, there are intriguing exceptions. In one study, mice previously infected with *Listeria monocytogenes* exhibited improved control of *L. major* following transfer of *Listeria*-pulsed dendritic cells (65). The authors proposed that memory CD8 T cells specific for *Listeria* were able to recognize and kill *Leishmania*-infected cells, though this mechanism was not formally demonstrated. As described above, more recent work has reinforced the concept that bystander activation is an important component of the CD8 T cell landscape in leishmaniasis, particularly in the skin. However, the extent to which these cells contribute to protection vs pathology likely depends on the nature of the co-infecting pathogen, the inflammatory milieu, and the presence of activating signals such as IL-15 or NKG2D ligands. As such, bystander CD8 T cells may represent a double-edged sword, capable of amplifying immune responses but also inflicting collateral damage in tissues already under stress.

### Inhibitory receptors, T cell exhaustion, and senescence

During chronic infections, persistent antigen exposure and inflammation can lead to functional impairments in T cells, often through mechanisms referred to as exhaustion or senescence (66). While these two states share some phenotypic overlap, including the upregulation of inhibitory receptors, they are distinct in origin and consequence. T cell exhaustion typically arises from prolonged antigen stimulation, resulting in a progressive loss of effector functions such as cytokine production and proliferation. Exhausted T cells often express inhibitory receptors like PD-1, CTLA-4, LAG-3, and TIM-3, and while they retain some functionality, their responsiveness is markedly diminished. In contrast, T cell senescence reflects a more terminal state associated with cellular aging, telomere shortening, DNA damage, and a pro-inflammatory secretory phenotype (67). Senescent T cells may retain cytotoxic capabilities and contribute to tissue pathology, but they are often resistant to proliferation and refractory to checkpoint blockade. Differentiating these states in *Leishmania* infection is critical, as they have different implications for both disease progression and potential therapeutic interventions.

**Fig 6:**
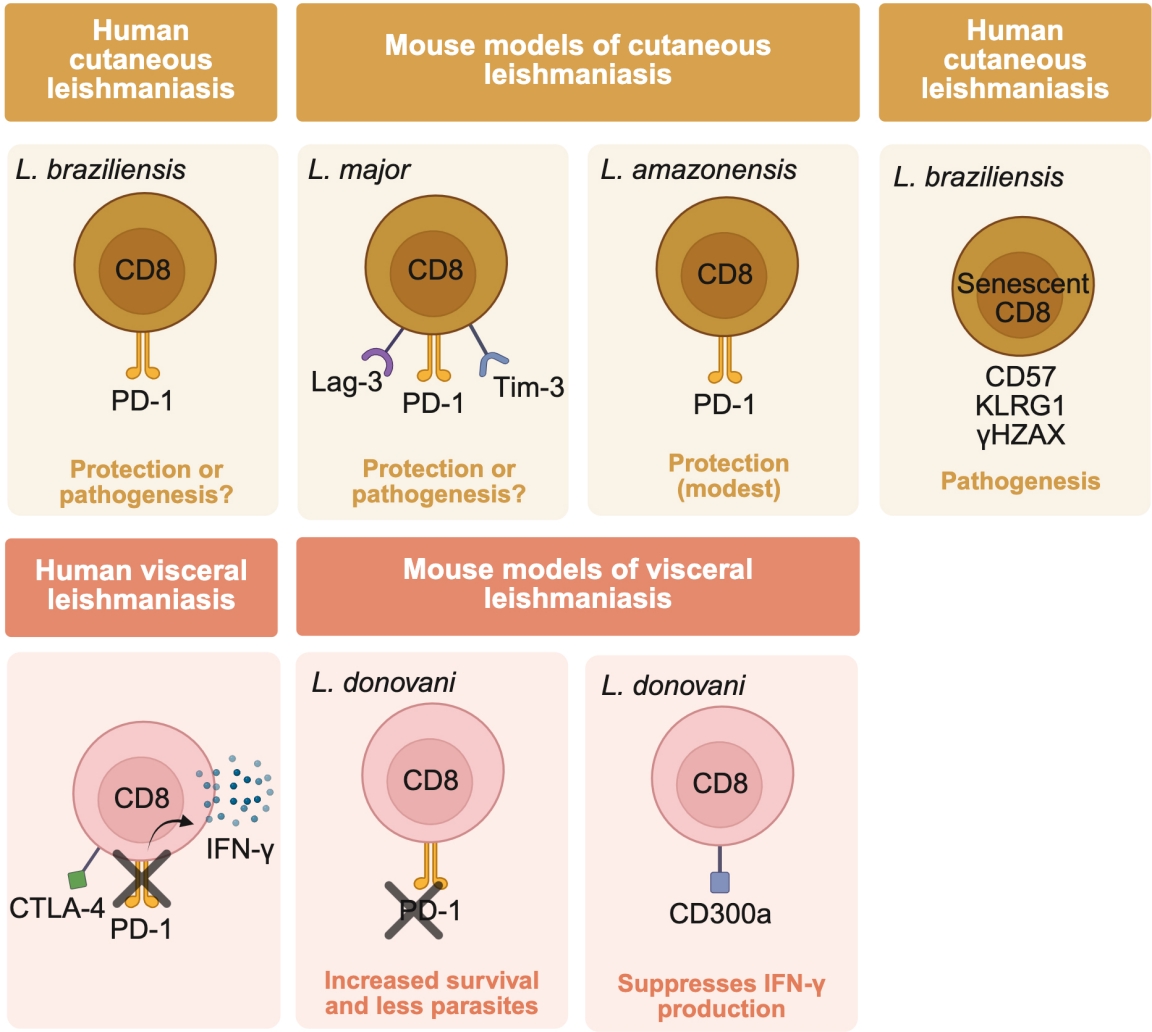
Exhaustion and senescence markers on CD8 T cells in leishmaniasis. The expression of inhibitory molecules such as PD-1, Tim-3, and Lag-3 is described in patients and mouse models of cutaneous leishmaniasis with various parasite species. However, it remains unclear what role these inhibitory markers, particularly PD-1, play in the pathologic or protective phenotype of CD8 T cells in cutaneous disease. In contrast, the inhibition of PD-1 on CD8 T cells during visceral disease in mice and humans increased the production of the protective cytokine IFN-γ and the survival of CD8 T cells. Other inhibitory markers, such as CTLA-4 and CD300a, are increased in visceral disease, but only the latter was shown to suppress IFN-γ production. Investigation into the role of senescence has been limited to cutaneous patients, with strong evidence suggesting a link between a senescent signature and cytotoxic, pathogenic CD8 T cells in the disease.

Inhibitory receptor expression has been documented in both blood and lesional T cells from patients with cutaneous leishmaniasis caused by *L. braziliensis* (Fig. 6). For example, PD-1 is expressed by CD8 T cells, although its expression does not correlate with lesion size, leading some to conclude that PD-1 may not be a major driver of pathology in this setting (68). In contrast, in cutaneous infection caused by *L. amazonensis* in mice, PD-1 or PD-L1 blockade increased IFN-γ production by CD8 T cells, though the impact on lesion size and parasite load was modest (69), suggesting partial functional recovery. In visceral leishmaniasis, PBMCs from patients stimulated with soluble *Leishmania* antigen produce more IFN-γ in the presence of PD-1 blockade, suggesting that inhibitory signaling constrains T cell function (70). Consistent with this, higher expression of PD-1 and CTLA-4 mRNA is observed in splenic aspirates compared to peripheral blood from visceral leishmaniasis patients, and isolated splenic CD8 T cells show elevated exhaustion markers (40). In mouse models of *L. donovani*, PD-1/PD-L1 blockade increased CD8 T cell survival and reduced parasite burden, though cytokine production was not fully restored (71).

Other inhibitory pathways are also immunosuppressive. CD300a, an inhibitory receptor that binds phosphatidylserine and phosphatidylethanolamine, is expressed on both myeloid and lymphoid cells (Fig. 6). In *L. donovani*-infected mice, CD300a deficiency led to increased IFN-γ production by both CD4 and CD8 T cells in the spleen, along with a significant reduction in parasite burden (72, 73). These findings suggest that the engagement of CD300a may dampen protective immunity and promote parasite persistence.

While it is still unknown how CD8 T cells express exhaustion markers in leishmaniasis, recent work suggests that the tissue microenvironment plays a role. Exposure to hypoxia and expression of the transcriptional repressor Blimp-1 are both known to promote the expression of co-inhibitory receptors associated with CD8 T cell exhaustion (74–78). Evidence from *L. major*-infected mice supports the presence of an exhausted-like phenotype in skin-infiltrating CD8 T cells. In one study, exhaustion-related gene signatures—defined initially in the context of chronic lymphocytic choriomeningitis virus infection for stem-like (CD101⁻Tim3⁻), transitory (CD101⁻Tim3^+^), and exhausted (CD101^+^Tim3^+^) PD-1^+^ CD8 T cell subsets—were compared with transcriptional profiles of CD8 T cells isolated from the lesions and draining lymph nodes of *L. major*-infected mice (25). CD8 T cells from lesions were enriched for all three exhaustion signatures compared to those from the lymph nodes. Elevated expression of canonical co-inhibitory receptor genes, such as *Pdcd1* (PD-1), *Lag3*, and *Havcr2* (Tim-3), was observed in lesional CD8 T cells, while other exhaustion-associated genes, including *Tox*, *Cx3cr1*, and *Tcf7*, did not differ between tissues. Protein-level analysis confirmed increased expression of PD-1, Lag-3, and Tim-3 on CD8 T cells in lesions. These findings suggest that a subset of CD8 T cells in *Leishmania*-infected skin expresses co-inhibitory receptors, consistent with a state of terminal differentiation or functional exhaustion. However, whether this expression translates into functional exhaustion and/or inhibits protective responses, similarly to *L. amazonensis* infection, is still unknown.

Senescent CD8 T cells, in contrast to exhausted cells, may retain or even enhance cytotoxic function despite being non-proliferative. In cutaneous leishmaniasis, the group of Gomes and colleagues has characterized a population of senescent CD8 T cells in patients infected with *L. braziliensis* (Fig. 6) (79–81). These cells are enriched in the peripheral blood and lesions, express classical senescence markers such as CD57 and KLRG1, display γH2AX positivity and shortened telomeres, and exhibit a pro-inflammatory, senescence-associated secretory phenotype-like phenotype. Importantly, the frequency of these cells correlates with lesion size (79, 81). Later work demonstrated that CD8 T cells, rather than senescent NK cells, were the dominant cytotoxic population within lesions, with enhanced skin-homing potential and expression of tissue-damaging effector molecules (79). A transcriptomic analysis of lesional tissue in 2021 (68) further confirmed the persistence of senescence- and inflammation-related gene signatures. However, these analyses were primarily based on bulk sequencing, and single-cell resolution was lacking. Thus, while the data strongly implicate senescent CD8 T cells in cutaneous immunopathology, definitive assignment of function to this subset awaits more granular approaches. Future work using single-cell and spatial transcriptomics will be essential to disentangle the contributions of senescent CD8 T cells from those of other inflammatory or cytotoxic populations in the skin.

## CONCLUSIONS AND OPEN QUESTIONS

CD8 T cells play a multifaceted role in leishmaniasis. Across studies, it is evident that their effector functions vary dramatically depending on the anatomical site, infecting *Leishmania* species, and disease form. In visceral leishmaniasis, CD8 T cells can support protective immunity through cytotoxic activity and cytokine production, particularly in lymphoid tissues. In contrast, in cutaneous disease, especially infections caused by *L. braziliensis*, cytotoxic CD8 T cells are more often associated with tissue destruction than parasite control. Although significant progress has been made in understanding their function, our knowledge of the antigen specificity, functional heterogeneity, and regulation of CD8 T cells in leishmaniasis remains limited.

Key questions remain unanswered. Despite great efforts (82–94), the antigens recognized by CD8 T cells during *Leishmania* infection have not been identified, making it difficult to study antigen-specific responses with precision. The role of distinct CD8 T cell subsets, such as tissue-resident memory cells and virtual memory T cells, remains largely unexplored in both human disease and experimental models. Furthermore, the contribution of bystander CD8 T cells, the dynamics of exhaustion vs senescence, and the plasticity of CD8 T cell phenotypes across different tissues and time points are not well understood.

In cutaneous diseases, CD8 T cells are exposed to a diverse array of microbial communities, including commensal viruses, bacteria, and fungi. These communities are dynamic and often shift during inflammatory conditions. In cutaneous leishmaniasis, for example, bacterial dysbiosis is a well-documented feature that influences disease outcomes (95–98). However, how these alterations in the microbiota shape the local microenvironment and impact CD8 T cell function remains poorly understood. Moreover, whether CD8 T cells specific for commensal or opportunistic microbes are present within lesions is an important and largely unexplored area of investigation.

Many of the human studies to date rely heavily on correlative data, with only a few providing mechanistic insights. The complexity of leishmaniasis, with its diverse clinical manifestations, multiple parasite species, and variable host responses, adds further layers of challenge to interpreting findings across models and settings. In human studies, technical limitations are compounded by logistical barriers: few field sites where leishmaniasis is endemic are equipped with the tools necessary to perform in-depth immunological studies. In experimental models, access to tools for studying antigen-specific CD8 T cells remains limited.

Moving forward, progress in this field will require both technological and conceptual advances. Single-cell and spatial technologies, antigen discovery platforms, and new models that reflect the heterogeneity of natural infection will be critical. A deeper understanding of CD8 T cell responses in leishmaniasis has the potential not only to clarify their role in protection and pathology but also to inform the design of next-generation vaccines and host-directed therapies.

## References

[1] Kaye P, Scott P. 2011. Leishmaniasis: complexity at the host-pathogen interface. Nat Rev Microbiol 9:604–615. doi:10.1038/nrmicro2608 21747391

[2] Scott P, Novais FO. 2016. Cutaneous leishmaniasis: immune responses in protection and pathogenesis. Nat Rev Immunol 16:581–592. doi:10.1038/nri.2016.72 27424773

[3] Sengupta R, Roy M, Dey NS, Kaye PM, Chatterjee M. 2023. Immune dysregulation and inflammation causing hypopigmentation in post kala-azar dermal leishmaniasis: partners in crime? Trends Parasitol 39:822–836. doi:10.1016/j.pt.2023.07.005 37586987

[4] Christo SN, Park SL, Mueller SN, Mackay LK. 2024. The multifaceted role of tissue-resident memory T cells. Annu Rev Immunol 42:317–345. doi:10.1146/annurev-immunol-101320-020220 38941605

[5] Bertholet S, Debrabant A, Afrin F, Caler E, Mendez S, Tabbara KS, Belkaid Y, Sacks DL. 2005. Antigen requirements for efficient priming of CD8+ T cells by Leishmania major-infected dendritic cells. Infect Immun 73:6620–6628. doi:10.1128/IAI.73.10.6620-6628.2005 16177338 PMC1230980

[6] Prickett S, Gray PM, Colpitts SL, Scott P, Kaye PM, Smith DF. 2006. In vivo recognition of ovalbumin expressed by transgenic Leishmania is determined by its subcellular localization. J Immunol 176:4826–4833. doi:10.4049/jimmunol.176.8.4826 16585577 PMC2695601

[7] Pamer EG, Sijts AJ, Villanueva MS, Busch DH, Vijh S. 1997. MHC class I antigen processing of Listeria monocytogenes proteins: implications for dominant and subdominant CTL responses. Immunol Rev 158:129–136. doi:10.1111/j.1600-065x.1997.tb00999.x 9314081

[8] Mazzaccaro RJ, Gedde M, Jensen ER, van Santen HM, Ploegh HL, Rock KL, Bloom BR. 1996. Major histocompatibility class I presentation of soluble antigen facilitated by Mycobacterium tuberculosis infection. Proc Natl Acad Sci USA 93:11786–11791. doi:10.1073/pnas.93.21.11786 8876215 PMC38136

[9] Bertholet S, Goldszmid R, Morrot A, Debrabant A, Afrin F, Collazo-Custodio C, Houde M, Desjardins M, Sher A, Sacks D. 2006. Leishmania antigens are presented to CD8+ T cells by a transporter associated with antigen processing-independent pathway in vitro and in vivo. J Immunol 177:3525–3533. doi:10.4049/jimmunol.177.6.3525 16951311

[10] Brosch S, Tenzer S, Akkad N, Lorenz B, Schild H, von Stebut E. 2012. Priming of Leishmania-reactive CD8+ T cells in vivo does not require LMP7-containing immunoproteasomes. J Invest Dermatol 132:1302–1305. doi:10.1038/jid.2011.454 22277939

[11] Ribeiro-Gomes FL, Romano A, Lee S, Roffê E, Peters NC, Debrabant A, Sacks D. 2015. Apoptotic cell clearance of Leishmania major-infected neutrophils by dendritic cells inhibits CD8. Cell Death Dis 6:e2018. doi:10.1038/cddis.2015.351 PMC4720886 26658192

[12] Khouili SC, Cook ECL, Hernández-García E, Martínez-López M, Conde-Garrosa R, Iborra S. 2020. SHP-1 regulates antigen cross-presentation and is exploited by leishmania to evade immunity. Cell Rep 33:108468. doi:10.1016/j.celrep.2020.108468 33264612

[13] Novais FO, Carvalho LP, Passos S, Roos DS, Carvalho EM, Scott P, Beiting DP. 2015. Genomic profiling of human Leishmania braziliensis lesions identifies transcriptional modules associated with cutaneous immunopathology. J Invest Dermatol 135:94–101. doi:10.1038/jid.2014.305 25036052 PMC4268311

[14] Rosas LE, Barbi J, Lu B, Fujiwara Y, Gerard C, Sanders VM, Satoskar AR. 2005. CXCR3-/- mice mount an efficient Th1 response but fail to control Leishmania major infection. Eur J Immunol 35:515–523. doi:10.1002/eji.200425422 15668916

[15] Majumder S, Bhattacharjee S, Paul Chowdhury B, Majumdar S. 2012. CXCL10 Is critical for the generation of protective CD8 T cell response induced by antigen pulsed CpG-ODN activated dendritic cells. PLoS One 7:e48727. doi:10.1371/journal.pone.0048727 23144947 PMC3492407

[16] Sacramento LA, Amorim CF, Lombana C, Scott P. 2023. CD8+ T cells require CCR5 expression to mediate immunopathology in cutaneous leishmaniasis. J Immunol 210:81. doi:10.4049/jimmunol.210.Supp.81.15

[17] Mukherjee S, Sengupta R, Mukhopadhyay D, Braun C, Mitra S, Roy S, Kanti Das N, Chatterjee U, von Stebut E, Chatterjee M. 2019. Impaired activation of lesional CD8^+^ T-cells is associated with enhanced expression of programmed death-1 in Indian post Kala-azar dermal leishmaniasis. Sci Rep 9:762. doi:10.1038/s41598-018-37144-y 30679687 PMC6345993

[18] Santos C da S, Boaventura V, Ribeiro Cardoso C, Tavares N, Lordelo MJ, Noronha A, Costa J, Borges VM, de Oliveira CI, Van Weyenbergh J, Barral A, Barral-Netto M, Brodskyn CI. 2013. CD8(+) granzyme B(+)-mediated tissue injury vs. CD4(+)IFNγ(+)-mediated parasite killing in human cutaneous leishmaniasis. J Invest Dermatol 133:1533–1540. doi:10.1038/jid.2013.4 23321919 PMC3667352

[19] Faria DR, Souza PEA, Durães FV, Carvalho EM, Gollob KJ, Machado PR, Dutra WO. 2009. Recruitment of CD8(+) T cells expressing granzyme A is associated with lesion progression in human cutaneous leishmaniasis. Parasite Immunol 31:432–439. doi:10.1111/j.1365-3024.2009.01125.x 19646207 PMC2764276

[20] Parkash V, Ashwin H, Dey S, Sadlova J, Vojtkova B, Van Bocxlaer K, Wiggins R, Thompson D, Dey NS, Jaffe CL, Schwartz E, Volf P, Lacey CJN, Layton AM, Kaye PM. 2024. Safety and reactogenicity of a controlled human infection model of sand fly-transmitted cutaneous leishmaniasis. Nat Med 30:3150–3162. doi:10.1038/s41591-024-03146-9 39095597 PMC11564116

[21] Belkaid Y, Von Stebut E, Mendez S, Lira R, Caler E, Bertholet S, Udey MC, Sacks D. 2002. CD8+ T cells are required for primary immunity in C57BL/6 mice following low-dose, intradermal challenge with Leishmania major. J Immunol 168:3992–4000. doi:10.4049/jimmunol.168.8.3992 11937556

[22] Uzonna JE, Joyce KL, Scott P. 2004. Low dose Leishmania major promotes a transient T helper cell type 2 response that is down-regulated by interferon gamma-producing CD8+ T cells. J Exp Med 199:1559–1566. doi:10.1084/jem.20040172 15184505 PMC2211781

[23] Herath S, Kropf P, Müller I. 2003. Cross-talk between CD8(+) and CD4(+) T cells in experimental cutaneous leishmaniasis: CD8(+) T cells are required for optimal IFN-gamma production by CD4(+) T cells. Parasite Immunol 25:559–567. doi:10.1111/j.0141-9838.2004.00668.x 15053777

[24] Novais FO, Wong AC, Villareal DO, Beiting DP, Scott P. 2018. CD8+ T cells lack local signals to produce IFN-γ in the skin during Leishmania infection. J Immunol 200:1737–1745. doi:10.4049/jimmunol.1701597 29367210 PMC6178231

[25] Fowler EA, Farias Amorim C, Mostacada K, Yan A, Amorim Sacramento L, Stanco RA, Hales ED, Varkey A, Zong W, Wu GD, de Oliveira CI, Collins PL, Novais FO. 2024. Neutrophil-mediated hypoxia drives pathogenic CD8+ T cell responses in cutaneous leishmaniasis. J Clin Invest 134:e177992. doi:10.1172/JCI177992 38833303 PMC11245163

[26] Cardoso TM, Machado Á, Costa DL, Carvalho LP, Queiroz A, Machado P, Scott P, Carvalho EM, Bacellar O. 2015. Protective and pathological functions of CD8+ T cells in Leishmania braziliensis infection. Infect Immun 83:898–906. doi:10.1128/IAI.02404-14 25534940 PMC4333467

[27] Nateghi Rostami M, Keshavarz H, Edalat R, Sarrafnejad A, Shahrestani T, Mahboudi F, Khamesipour A. 2010. CD8+ T cells as a source of IFN-γ production in human cutaneous leishmaniasis. PLoS Negl Trop Dis 4:e845. doi:10.1371/journal.pntd.0000845 20967288 PMC2953482

[28] Boaventura VS, Santos CS, Cardoso CR, de Andrade J, Dos Santos WLC, Clarêncio J, Silva JS, Borges VM, Barral‐Netto M, Brodskyn CI, Barral A. 2010. Human mucosal leishmaniasis: neutrophils infiltrate areas of tissue damage that express high levels of Th17‐related cytokines. Eur J Immunol 40:2830–2836. doi:10.1002/eji.200940115 20812234

[29] Novais FO, Carvalho LP, Graff JW, Beiting DP, Ruthel G, Roos DS, Betts MR, Goldschmidt MH, Wilson ME, de Oliveira CI, Scott P. 2013. Cytotoxic T cells mediate pathology and metastasis in cutaneous leishmaniasis. PLoS Pathog 9:e1003504. doi:10.1371/journal.ppat.1003504 23874205 PMC3715507

[30] Voskoboinik I, Whisstock JC, Trapani JA. 2015. Perforin and granzymes: function, dysfunction and human pathology. Nat Rev Immunol 15:388–400. doi:10.1038/nri3839 25998963

[31] Chung W-H, Hung S-I, Yang J-Y, Su S-C, Huang S-P, Wei C-Y, Chin S-W, Chiou C-C, Chu S-C, Ho H-C, Yang C-H, Lu C-F, Wu J-Y, Liao Y-D, Chen Y-T. 2008. Granulysin is a key mediator for disseminated keratinocyte death in Stevens-Johnson syndrome and toxic epidermal necrolysis. Nat Med 14:1343–1350. doi:10.1038/nm.1884 19029983

[32] van den Boorn JG, Konijnenberg D, Dellemijn TAM, van der Veen JPW, Bos JD, Melief CJM, Vyth-Dreese FA, Luiten RM. 2009. Autoimmune destruction of skin melanocytes by perilesional T cells from vitiligo patients. J Invest Dermatol 129:2220–2232. doi:10.1038/jid.2009.32 19242513

[33] Zloza A, Lyons GE, Chlewicki LK, Kohlhapp FJ, O’Sullivan JA, Lacek AT, Moore TV, Jagoda MC, Kumar V, Guevara-Patiño JA. 2011. Engagement of NK receptor NKG2D, but not 2B4, results in self-reactive CD8+ T cells and autoimmune vitiligo. Autoimmunity 44:599–606. doi:10.3109/08916934.2011.593599 21913803

[34] Xing L, Dai Z, Jabbari A, Cerise JE, Higgins CA, Gong W, de Jong A, Harel S, DeStefano GM, Rothman L, Singh P, Petukhova L, Mackay-Wiggan J, Christiano AM, Clynes R. 2014. Alopecia areata is driven by cytotoxic T lymphocytes and is reversed by JAK inhibition. Nat Med 20:1043–1049. doi:10.1038/nm.3645 25129481 PMC4362521

[35] Jacquemin C, Martins C, Lucchese F, Thiolat D, Taieb A, Seneschal J, Boniface K. 2020. NKG2D defines a subset of skin effector memory CD8 T cells with proinflammatory functions in vitiligo. J Invest Dermatol 140:1143–1153. doi:10.1016/j.jid.2019.11.013 31877315

[36] Hiroyasu S, Zeglinski MR, Zhao H, Pawluk MA, Turner CT, Kasprick A, Tateishi C, Nishie W, Burleigh A, Lennox PA, Van Laeken N, Carr NJ, Petersen F, Crawford RI, Shimizu H, Tsuruta D, Ludwig RJ, Granville DJ. 2021. Granzyme B inhibition reduces disease severity in autoimmune blistering diseases. Nat Commun 12:302. doi:10.1038/s41467-020-20604-3 33436591 PMC7804321

[37] Egui A, Ledesma D, Pérez-Antón E, Montoya A, Gómez I, Robledo SM, Infante JJ, Vélez ID, López MC, Thomas MC. 2018. Phenotypic and functional profiles of antigen-specific CD4+ and CD8+ T cells associated with infection control in patients with cutaneous leishmaniasis. Front Cell Infect Microbiol 8:393. doi:10.3389/fcimb.2018.00393 30510917 PMC6252334

[38] Hernández-Ruiz J, Salaiza-Suazo N, Carrada G, Escoto S, Ruiz-Remigio A, Rosenstein Y, Zentella A, Becker I. 2010. CD8 cells of patients with diffuse cutaneous leishmaniasis display functional exhaustion: the latter is reversed, in vitro, by TLR2 agonists. PLoS Negl Trop Dis 4:e871. doi:10.1371/journal.pntd.0000871 21072232 PMC2970528

[39] Faria DR, Gollob KJ, Barbosa J Jr, Schriefer A, Machado PRL, Lessa H, Carvalho LP, Romano-Silva MA, de Jesus AR, Carvalho EM, Dutra WO. 2005. Decreased in situ expression of interleukin-10 receptor is correlated with the exacerbated inflammatory and cytotoxic responses observed in mucosal leishmaniasis. Infect Immun 73:7853–7859. doi:10.1128/IAI.73.12.7853-7859.2005 16299275 PMC1307048

[40] Gautam S, Kumar R, Singh N, Singh AK, Rai M, Sacks D, Sundar S, Nylén S. 2014. CD8 T cell exhaustion in human visceral leishmaniasis. J Infect Dis 209:290–299. doi:10.1093/infdis/jit401 23922369 PMC3873784

[41] Amorim CF, Novais FO, Nguyen BT, Misic AM, Carvalho LP, Carvalho EM, Beiting DP, Scott P. 2019. Variable gene expression and parasite load predict treatment outcome in cutaneous leishmaniasis. Sci Transl Med 11:eaax4204. doi:10.1126/scitranslmed.aax4204 31748229 PMC7068779

[42] Novais FO, Nguyen BT, Scott P. 2021. Granzyme B inhibition by tofacitinib blocks the pathology induced by CD8 T cells in cutaneous leishmaniasis. J Invest Dermatol 141:575–585. doi:10.1016/j.jid.2020.07.011 32738245 PMC7855313

[43] Novais FO, Carvalho AM, Clark ML, Carvalho LP, Beiting DP, Brodsky IE, Carvalho EM, Scott P. 2017. CD8+ T cell cytotoxicity mediates pathology in the skin by inflammasome activation and IL-1β production. PLoS Pathog 13:e1006196. doi:10.1371/journal.ppat.1006196 28192528 PMC5325592

[44] Fowler EA, Amorim Sacramento L, Bowman BA, Lee B, Lio C-W, Dong Y-D, Spicer JA, Trapani JA, Novais FO. 2025. Hypoxia and IL-15 cooperate to induce perforin expression by CD8 T cells and promote damage to the skin in murine cutaneous leishmaniasis. J Invest Dermatol:S0022-202X(25)00479-8. doi:10.1016/j.jid.2025.04.029 PMC12802810 40373956

[45] Lima-Junior DS, Costa DL, Carregaro V, Cunha LD, Silva ALN, Mineo TWP, Gutierrez FRS, Bellio M, Bortoluci KR, Flavell RA, Bozza MT, Silva JS, Zamboni DS. 2013. Inflammasome-derived IL-1β production induces nitric oxide-mediated resistance to Leishmania. Nat Med 19:909–915. doi:10.1038/nm.3221 23749230

[46] Belkaid Y, Hoffmann KF, Mendez S, Kamhawi S, Udey MC, Wynn TA, Sacks DL. 2001. The role of interleukin (IL)-10 in the persistence of Leishmania major in the skin after healing and the therapeutic potential of anti-IL-10 receptor antibody for sterile cure. J Exp Med 194:1497–1506. doi:10.1084/jem.194.10.1497 11714756 PMC2193677

[47] Okwor IB, Jia P, Mou Z, Onyilagha C, Uzonna JE. 2014. CD8+ T cells are preferentially activated during primary low dose leishmania major infection but are completely dispensable during secondary anti-Leishmania immunity. PLoS Negl Trop Dis 8:e3300. doi:10.1371/journal.pntd.0003300 25412267 PMC4238992

[48] Rhee EG, Mendez S, Shah JA, Wu C, Kirman JR, Turon TN, Davey DF, Davis H, Klinman DM, Coler RN, Sacks DL, Seder RA. 2002. Vaccination with heat-killed leishmania antigen or recombinant leishmanial protein and CpG oligodeoxynucleotides induces long-term memory CD4+ and CD8+ T cell responses and protection against leishmania major infection. J Exp Med 195:1565–1573. doi:10.1084/jem.20020147 12070284 PMC2193566

[49] Colmenares M, Kima PE, Samoff E, Soong L, McMahon-Pratt D. 2003. Perforin and gamma interferon are critical CD8+ T-cell-mediated responses in vaccine-induced immunity against Leishmania amazonensis infection. Infect Immun 71:3172–3182. doi:10.1128/IAI.71.6.3172-3182.2003 12761096 PMC155724

[50] Stäger S, Alexander J, Kirby AC, Botto M, Rooijen NV, Smith DF, Brombacher F, Kaye PM. 2003. Natural antibodies and complement are endogenous adjuvants for vaccine-induced CD8+ T-cell responses. Nat Med 9:1287–1292. doi:10.1038/nm933 14502281

[51] Basu R, Bhaumik S, Haldar AK, Naskar K, De T, Dana SK, Walden P, Roy S. 2007. Hybrid cell vaccination resolves Leishmania donovani infection by eliciting a strong CD8+ cytotoxic T-lymphocyte response with concomitant suppression of interleukin-10 (IL-10) but not IL-4 or IL-13. Infect Immun 75:5956–5966. doi:10.1128/IAI.00944-07 17908806 PMC2168357

[52] Kronenberg K, Brosch S, Butsch F, Tada Y, Shibagaki N, Udey MC, von Stebut E. 2010. Vaccination with TAT-antigen fusion protein induces protective, CD8(+) T cell-mediated immunity against Leishmania major. J Invest Dermatol 130:2602–2610. doi:10.1038/jid.2010.171 20574442 PMC6999697

[53] Jayakumar A, Castilho TM, Park E, Goldsmith-Pestana K, Blackwell JM, McMahon-Pratt D. 2011. TLR1/2 activation during heterologous prime-boost vaccination (DNA-MVA) enhances CD8+ T Cell responses providing protection against Leishmania (Viannia). PLoS Negl Trop Dis 5:e1204. doi:10.1371/journal.pntd.0001204 21695103 PMC3114751

[54] Nico D, Gomes DC, Alves-Silva MV, Freitas EO, Morrot A, Bahia D, Palatnik M, Rodrigues MM, Palatnik-de-Sousa CB. 2014. Cross-protective immunity to Leishmania amazonensis is mediated by CD4+ and CD8+ epitopes of Leishmania donovani nucleoside hydrolase terminal domains. Front Immunol 5:189. doi:10.3389/fimmu.2014.00189 24822054 PMC4013483

[55] Bogdan C, Islam N-A-K, Barinberg D, Soulat D, Schleicher U, Rai B. 2024. The immunomicrotope of Leishmania control and persistence. Trends Parasitol 40:788–804. doi:10.1016/j.pt.2024.07.013 39174373

[56] Mahnke A, Meier RJ, Schatz V, Hofmann J, Castiglione K, Schleicher U, Wolfbeis OS, Bogdan C, Jantsch J. 2014. Hypoxia in Leishmania major skin lesions impairs the NO-dependent leishmanicidal activity of macrophages. J Invest Dermatol 134:2339–2346. doi:10.1038/jid.2014.121 24583949

[57] Hammami A, Abidin BM, Heinonen KM, Stäger S. 2018. HIF-1α hampers dendritic cell function and Th1 generation during chronic visceral leishmaniasis. Sci Rep 8:3500. doi:10.1038/s41598-018-21891-z 29472618 PMC5823892

[58] Sacramento LA, Farias Amorim C, Campos TM, Saldanha M, Arruda S, Carvalho LP, Beiting DP, Carvalho EM, Novais FO, Scott P. 2023. NKG2D promotes CD8 T cell-mediated cytotoxicity and is associated with treatment failure in human cutaneous leishmaniasis. PLoS Negl Trop Dis 17:e0011552. doi:10.1371/journal.pntd.0011552 37603573 PMC10470908

[59] Figliuolo VR, Chaves SP, Savio LEB, Thorstenberg MLP, Machado Salles É, Takiya CM, D’Império-Lima MR, de Matos Guedes HL, Rossi-Bergmann B, Coutinho-Silva R. 2017. The role of the P2X7 receptor in murine cutaneous leishmaniasis: aspects of inflammation and parasite control. Purinergic Signal 13:143–152. doi:10.1007/s11302-016-9544-1 27866341 PMC5432475

[60] Norsworthy NB, Sun J, Elnaiem D, Lanzaro G, Soong L. 2004. Sand fly saliva enhances Leishmania amazonensis infection by modulating interleukin-10 production. Infect Immun 72:1240–1247. doi:10.1128/IAI.72.3.1240-1247.2004 14977924 PMC356033

[61] Polley R, Stager S, Prickett S, Maroof A, Zubairi S, Smith DF, Kaye PM. 2006. Adoptive immunotherapy against experimental visceral leishmaniasis with CD8+ T cells requires the presence of cognate antigen. Infect Immun 74:773–776. doi:10.1128/IAI.74.1.773-776.2006 16369038 PMC1346645

[62] Crosby EJ, Goldschmidt MH, Wherry EJ, Scott P. 2014. Engagement of NKG2D on bystander memory CD8 T cells promotes increased immunopathology following Leishmania major infection. PLoS Pathog 10:e1003970. doi:10.1371/journal.ppat.1003970 24586170 PMC3937277

[63] Crosby EJ, Clark M, Novais FO, Wherry EJ, Scott P. 2015. Lymphocytic choriomeningitis virus expands a population of NKG2D+CD8+ T cells that exacerbates disease in mice coinfected with Leishmania major. J Immunol 195:3301–3310. doi:10.4049/jimmunol.1500855 26290604 PMC4575880

[64] Da-Cruz AM, Oliveira-Neto MP, Bertho AL, Mendes-Aguiar CO, Coutinho SG. 2010. T cells specific to leishmania and other nonrelated microbial antigens can migrate to human leishmaniasis skin lesions. J Invest Dermatol 130:1329–1336. doi:10.1038/jid.2009.428 20107484

[65] Narni-Mancinelli E, Campisi L, Bassand D, Cazareth J, Gounon P, Glaichenhaus N, Lauvau G. 2007. Memory CD8+ T cells mediate antibacterial immunity via CCL3 activation of TNF/ROI+ phagocytes. J Exp Med 204:2075–2087. doi:10.1084/jem.20070204 17698589 PMC2118695

[66] Rausch L, Kallies A. 2025. Molecular mechanisms governing CD8 T cell differentiation and checkpoint inhibitor response in cancer. Annu Rev Immunol 43:515–543. doi:10.1146/annurev-immunol-082223-044122 40279308

[67] Wu H, Li J, Zhang Z, Zhang Y. 2024. Characteristics and mechanisms of T-cell senescence: a potential target for cancer immunotherapy. Eur J Immunol 54:e2451093. doi:10.1002/eji.202451093 39107923

[68] Garcia de Moura R, Covre LP, Fantecelle CH, Gajardo VAT, Cunha CB, Stringari LL, Belew AT, Daniel CB, Zeidler SVV, Tadokoro CE, de Matos Guedes HL, Zanotti RL, Mosser D, Falqueto A, Akbar AN, Gomes DCO. 2021. PD-1 blockade modulates functional activities of exhausted-like t cell in patients with cutaneous leishmaniasis. Front Immunol 12:632667. doi:10.3389/fimmu.2021.632667 33767700 PMC7985249

[69] da Fonseca-Martins AM, Ramos TD, Pratti JES, Firmino-Cruz L, Gomes DCO, Soong L, Saraiva EM, de Matos Guedes HL. 2019. Immunotherapy using anti-PD-1 and anti-PD-L1 in Leishmania amazonensis-infected BALB/c mice reduce parasite load. Sci Rep 9:20275. doi:10.1038/s41598-019-56336-8 31889072 PMC6937231

[70] Takele Y, Adem E, Franssen SU, Womersley R, Kaforou M, Levin M, Müller I, Cotton JA, Kropf P. 2022. Impaired in vitro Interferon-γ production in patients with visceral leishmaniasis is improved by inhibition of PD1/PDL-1 ligation. PLoS Negl Trop Dis 16:e0010544. doi:10.1371/journal.pntd.0010544 35749568 PMC9262188

[71] Joshi T, Rodriguez S, Perovic V, Cockburn IA, Stäger S. 2009. B7-H1 blockade increases survival of dysfunctional CD8(+) T cells and confers protection against Leishmania donovani infections. PLoS Pathog 5:e1000431. doi:10.1371/journal.ppat.1000431 19436710 PMC2674929

[72] Anand A, Singh R, Saini S, Mahapatra B, Singh A, Singh S, Singh RK. 2023. Leishmania donovani induces CD300a expression to dampen effector properties of CD11c+ dendritic and antigen activated CD8+ T cells. Acta Trop 239:106826. doi:10.1016/j.actatropica.2023.106826 36610528

[73] Singh R, Anand A, Rawat AK, Saini S, Mahapatra B, Singh NK, Mishra AK, Singh S, Singh N, Kishore D, Kumar V, Das P, Singh RK. 2021. CD300a receptor blocking enhances early clearance of Leishmania donovani from its mammalian host through modulation of effector functions of phagocytic and antigen experienced T cells. Front Immunol 12:793611. doi:10.3389/fimmu.2021.793611 35116028 PMC8803664

[74] Shin H, Blackburn SD, Intlekofer AM, Kao C, Angelosanto JM, Reiner SL, Wherry EJ. 2009. A role for the transcriptional repressor blimp-1 in CD8+ T cell exhaustion during chronic viral infection. Immunity 31:309–320. doi:10.1016/j.immuni.2009.06.019 19664943 PMC2747257

[75] Zhu L, Kong Y, Zhang J, Claxton DF, Ehmann WC, Rybka WB, Palmisiano ND, Wang M, Jia B, Bayerl M, Schell TD, Hohl RJ, Zeng H, Zheng H. 2017. Blimp-1 impairs T cell function via upregulation of TIGIT and PD-1 in patients with acute myeloid leukemia. J Hematol Oncol 10:124. doi:10.1186/s13045-017-0486-z 28629373 PMC5477125

[76] Scharping NE, Rivadeneira DB, Menk AV, Vignali PDA, Ford BR, Rittenhouse NL, Peralta R, Wang Y, Wang Y, DePeaux K, Poholek AC, Delgoffe GM. 2021. Mitochondrial stress induced by continuous stimulation under hypoxia rapidly drives T cell exhaustion. Nat Immunol 22:205–215. doi:10.1038/s41590-020-00834-9 33398183 PMC7971090

[77] Bannoud N, Dalotto-Moreno T, Kindgard L, García PA, Blidner AG, Mariño KV, Rabinovich GA, Croci DO. 2021 Hypoxia supports differentiation of terminally exhausted CD8 T cells. Front Immunol 12. doi:10.3389/fimmu.2021.660944 PMC8137905 34025660

[78] Ford BR, Vignali PDA, Rittenhouse NL, Scharping NE, Peralta R, Lontos K, Frisch AT, Delgoffe GM, Poholek AC. 2022. Tumor microenvironmental signals reshape chromatin landscapes to limit the functional potential of exhausted T cells. Sci Immunol 7. doi:10.1126/sciimmunol.abj9123 PMC9851604 35930654

[79] Covre LP, Devine OP, Garcia de Moura R, Vukmanovic-Stejic M, Dietze R, Ribeiro-Rodrigues R, Guedes HLDM, Lubiana Zanotti R, Falqueto A, Akbar AN, Gomes DCO. 2020. Compartmentalized cytotoxic immune response leads to distinct pathogenic roles of natural killer and senescent CD8^+^ T cells in human cutaneous leishmaniasis. Immunology 159:429–440. doi:10.1111/imm.13173 31925782 PMC7078002

[80] Fantecelle CH, Covre LP, Garcia de Moura R, Guedes H de M, Amorim CF, Scott P, Mosser D, Falqueto A, Akbar AN, Gomes DCO. 2021. Transcriptomic landscape of skin lesions in cutaneous leishmaniasis reveals a strong CD8^+^ T cell immunosenescence signature linked to immunopathology. Immunology 164:754–765. doi:10.1111/imm.13410 34432883 PMC8561102

[81] Covre LP, Martins RF, Devine OP, Chambers ES, Vukmanovic-Stejic M, Silva JA, Dietze R, Rodrigues RR, de Matos Guedes HL, Falqueto A, Akbar AN, Gomes DCO. 2018 Circulating senescent T cells are linked to systemic inflammation and lesion size during human cutaneous leishmaniasis. Front Immunol 9. doi:10.3389/fimmu.2018.03001 PMC6328442 30662437

[82] Dikhit MR, Das S, Mahantesh V, Kumar A, Singh AK, Dehury B, Rout AK, Ali V, Sahoo GC, Topno RK, Pandey K, Das VNR, Bimal S, Das P. 2018 The potential HLA Class I-restricted epitopes derived from LeIF and TSA of Leishmania donovani evoke anti-leishmania CD8+ T lymphocyte response. Sci Rep 8. doi:10.1038/s41598-018-32040-x PMC6154976 30242172

[83] Resende DM, Caetano BC, Dutra MS, Penido MLO, Abrantes CF, Verly RM, Resende JM, Piló-Veloso D, Rezende SA, Bruna-Romero O, Fernandes AP, Gazzinelli RT. 2008. Epitope mapping and protective immunity elicited by adenovirus expressing the Leishmania amastigote specific A2 antigen: correlation with IFN-γ and cytolytic activity by CD8+ T cells. Vaccine (Auckland) 26:4585–4593. doi:10.1016/j.vaccine.2008.05.091 18588933

[84] Amit A, Dikhit MR, Singh AK, Venkateshwaran T, Das VNR, Das P, Bimal S. 2017. Immuno-informatics based approaches to identify CD8+ T cell epitopes within the Leishmania donovani 3-ectonucleotidase in cured visceral leishmaniasis subjects. Microbes Infect 19:358–369. doi:10.1016/j.micinf.2017.03.002 28373107

[85] Sabur A, Bhowmick S, Chhajer R, Ejazi SA, Didwania N, Asad M, Bhattacharyya A, Sinha U, Ali N. 2018 Liposomal elongation factor-1α triggers effector CD4 and CD8 T cells for induction of long-lasting protective immunity against visceral leishmaniasis. Front Immunol 9. doi:10.3389/fimmu.2018.00018 PMC5797590 29441060

[86] Dias DS, Ribeiro PAF, Martins VT, Lage DP, Costa LE, Chávez-Fumagalli MA, Ramos FF, Santos TTO, Ludolf F, Oliveira JS, Mendes TAO, Silva ES, Galdino AS, Duarte MC, Roatt BM, Menezes-Souza D, Teixeira AL, Coelho EAF. 2018. Vaccination with a CD4+ and CD8+ T-cell epitopes-based recombinant chimeric protein derived from Leishmania infantum proteins confers protective immunity against visceral leishmaniasis. Transl Res 200:18–34. doi:10.1016/j.trsl.2018.05.001 29908151

[87] RFRRFRMartins VT, Duarte MC, Lage DP, Costa LE, Carvalho A, Mendes TAO, Roatt BM, Menezes-Souza D, Soto M, Coelho EAF. 2017. A recombinant chimeric protein composed of human and mice-specific CD4+ and CD8+ T-cell epitopes protects against visceral leishmaniasis. Parasite Immunol 39. doi:10.1111/pim.12359 27593711

[88] de Oliveira BC, da Silva AA, de Andrade Cavalcante MK, de Brito MEF, de Castro MCAB, de Medeiros VLS, de Freitas e Silva R, Pereira VRA. 2023. Central and effector memory human CD4+ and CD8+ T cells during cutaneous leishmaniasis and after in vitro stimulation with leishmania (viannia) braziliensis epitopes. Vaccines (Basel) 11:158. doi:10.3390/vaccines11010158 36680003 PMC9861845

[89] Athanasiou E, Agallou M, Tastsoglou S, Kammona O, Hatzigeorgiou A, Kiparissides C, Karagouni E. 2017. A poly(Lactic-co-Glycolic) acid nanovaccine based on chimeric peptides from different leishmania infantum proteins induces dendritic cells maturation and promotes peptide-specific IFNγ-producing CD8+ T cells essential for the protection against experimental visceral leishmaniasis. Front Immunol 8:684. doi:10.3389/fimmu.2017.00684 28659922 PMC5468442

[90] e Silva R de F, de Oliveira BC, da Silva AA, Brelaz de Castro MCA, Ferreira LFGR, Hernandes MZ, de Brito MEF, de-Melo-Neto OP, Rezende AM, Pereira VRA. 2019. Immunogenicity of potential CD4+ and CD8+ T cell epitopes derived from the proteome of Leishmania braziliensis. Front Immunol 10:3145. doi:10.3389/fimmu.2019.03145 32117204 PMC7033680

[91] Duarte A, Queiroz ATL, Tosta R, Carvalho AM, Barbosa CH, Bellio M, de Oliveira CI, Barral-Netto M. 2015. Prediction of CD8+ epitopes in Leishmania braziliensis proteins using epibot: in silico search and in vivo validation. PLoS One 10:e0124786. doi:10.1371/journal.pone.0124786 25905908 PMC4407964

[92] Agallou M, Athanasiou E, Koutsoni O, Dotsika E, Karagouni E. 2014. Experimental validation of multi-epitope peptides including promising MHC class I- and II-restricted epitopes of four known Leishmania infantum proteins. Front Immunol 5:268. doi:10.3389/fimmu.2014.00268 24959167 PMC4051127

[93] Guerfali FZ, Ben-Abdallah H, Sghaier RM, Ben-Aissa K, Mkannez G, Attia H, Laouini D. 2009. An in silico immunological approach for prediction of CD8+ T cell epitopes of Leishmania major proteins in susceptible BALB/c and resistant C57BL/6 murine models of infection. Infect Genet Evol 9:344–350. doi:10.1016/j.meegid.2008.02.011 18420466

[94] Barreto AS, Franca MNF, Dos Reis T, Silva J, Dos Santos PL, Oliveira FA, Silva AM, Magalhaes LS, Secco DA, Andrade MAF, Porto LC, Rosa DS, Cavalcante RCM, Corrêa CB, Sidney J, Sette A, Almeida RP, Palatnik-de-Sousa CB. 2025. Design and development of highly conserved, HLA-promiscuous T cell multiepitope vaccines against human visceral leishmaniasis. Front Immunol 16:1540537. doi:10.3389/fimmu.2025.1540537 40230841 PMC11994619

[95] Gimblet C, Meisel JS, Loesche MA, Cole SD, Horwinski J, Novais FO, Misic AM, Bradley CW, Beiting DP, Rankin SC, Carvalho LP, Carvalho EM, Scott P, Grice EA. 2017. Cutaneous leishmaniasis induces a transmissible dysbiotic skin microbiota that promotes skin inflammation. Cell Host Microbe 22:13–24. doi:10.1016/j.chom.2017.06.006 28669672 PMC5555377

[96] Amorim CF, Lovins V, Singh TP, Novais FO, Harris J, Carvalho LP, Carvalho EM, Beiting DP, Grice EA, Scott P. 2023. Staphylococcus aureus promotes increase IL-1 mediated immunopathology and delayed healing in cutaneous leishmaniasis. J Immunol 210:81. doi:10.4049/jimmunol.210.Supp.81.03

[97] Singh TP, Carvalho AM, Sacramento LA, Grice EA, Scott P. 2021. Microbiota instruct IL-17A-producing innate lymphoid cells to promote skin inflammation in cutaneous leishmaniasis. PLoS Pathog 17:e1009693. doi:10.1371/journal.ppat.1009693 34699567 PMC8570469

[98] Salgado VR, Queiroz AT de, Sanabani SS, Oliveira CI de, Carvalho EM, Costa JM, Barral-Netto M, Barral A. 2016. The microbiological signature of human cutaneous leishmaniasis lesions exhibits restricted bacterial diversity compared to healthy skin. Mem Inst Oswaldo Cruz 111:241–251. doi:10.1590/0074-02760150436 27074253 PMC4830113

